# Effect of Laser Shock Peening on the Passivation Behavior of Subtractively and Additively Manufactured Ti–6Al–4V Alloys in pH 2 Buffer Solution

**DOI:** 10.3390/ma19071432

**Published:** 2026-04-03

**Authors:** JuHee Lee, Jan Kaufman, Martin Divoký, Tomáš Mocek, Jan Brajer, HeeJin Jang

**Affiliations:** 1 Department of Advanced Materials Engineering, Chosun University, 309 Pilmundaero, Dong-gu, Gwangju 61452, Republic of Korea; 2 HiLASE Centre, Institute of Physics of the Czech Academy of Sciences, Za Radnicí 828, 252 41 Dolní Břežany, Czech Republic; jan.kaufman@hilase.cz (J.K.);

**Keywords:** corrosion, Mott–Schottky, Ti–6Al–4V, additive manufacturing, laser shock peening

## Abstract

The effects of laser shock peening (LSP) on subtractively manufactured (SM) and additively manufactured (AM) Ti–6Al–4V alloys in pH 2 buffer solution were investigated. LSP increased the surface roughness from 0.25 ± 0.05 μm to 0.6 ± 0.1 μm, raised Vickers hardness by 12–16%, and introduced compressive residual stresses of 400–950 MPa. Microstructural analysis indicated that LSP promoted β-phase formation and grain refinement in SM alloys, while reducing the α′-phase fraction in AM alloys. Electrochemical testing revealed that all LSP-treated specimens exhibited active–passive transitions, unlike the stable passive response of unpeened samples. The corrosion rate (*i*_corr_) decreased from approximately 5 × 10^−6^ to 1 × 10^−6^ A·cm^−2^ after LSP. During 24 h potentiostatic polarization at 1.3 *V*_SCE_, the passive current density stabilized at 10^−8^–10^−7^ A·cm^−2^, with LSP AM specimens exhibiting values approximately twice those of their unpeened counterparts. Mott–Schottky analysis confirmed that the donor density (*N*_D_) in the SM alloy changed negligibly after LSP, indicating a stable passive alloy. In contrast, the *N*_D_ for the AM alloy increased from 1 × 10^19^ to 3 × 10^19^ cm^−3^, suggesting an oxygen-vacancy-rich, less stable passive film. Overall, LSP reduces the corrosion rate primarily through the introduction of compressive residual stress but may impair the long-term passive-film stability of AM Ti–6Al–4V owing to defect generation. In contrast, the SM alloy maintains passive-film stability under identical treatment conditions.

## 1. Introduction

Additive manufacturing (AM), also referred to as three-dimensional (3D) printing [1], is a promising technology for customizable and smart production, offering benefits such as the ability to manufacture complex shapes inaccessible to conventional techniques and reduced material costs. However, AM processes suffer from various problems, such as residual stresses, solidification defects, and uneven surface conditions arising from rapid temperature changes [2,3]. Post-processing and process-optimization strategies are typically required to mitigate these issues.

Ti alloys are widely used in the aerospace and biomedical industries owing to their low weight, excellent mechanical properties, and high corrosion resistance [4,5,6,7]. Notably, the mechanical performance of Ti alloys is strongly governed by microstructural features such as grain size, dislocation density, and interfacial characteristics [8]. Because of their high cost and difficulty in machining, Ti alloys are particularly suitable for AM processing [9]. Accordingly, various researchers have focused on the material properties of Ti alloys subjected to AM [10,11,12,13].

In addition, Ti and its alloys are passive metals that form a thin protective TiO_2_ film on their surfaces [14]. As in the case of other passive alloys, exposure to halogen ions can lead to localized breakdown of the passive film, resulting in localized corrosion and stress corrosion cracking (SCC) [15,16,17,18]. Several studies have reported that Ti alloys fabricated using AM are less corrosion resistant than those fabricated using subtractive manufacturing (SM) employing conventional casting and forging techniques [19,20,21,22]. This inferior resistance is largely attributable to the growth of stress corrosion cracks, promoted by the presence of pores and voids [21], or the tensile residual stresses introduced during the AM process. Therefore, numerous engineering approaches have been explored to eliminate pores, voids, and residual stresses in AM alloys [23,24,25,26].

Several mechanical surface modification techniques, including conventional shot peening (SP) [27], ultrasonic shot peening (USP) [28,29] and ultrasonic nanocrystal surface modification (UNSM) [30,31] have been investigated to enhance the mechanical and corrosion properties of metallic materials. These techniques induce compressive residual stresses and grain refinement near the surface, improving fatigue performance, wear resistance [32,33], pitting resistance [34,35], hardness, and corrosion resistance in both stainless steels and Ti alloys [36,37,38]. However, such surface modification processes involve repeated mechanical impacts or ultrasonic vibrations, which are often accompanied by localized temperature rises and potential surface contamination, making it difficult to completely exclude thermal or environmental effects during processing.

In contrast, laser shock peening (LSP) [39,40,41] employs high-intensity laser-induced shock waves to generate compressive residual stresses that can extend several hundred micrometers beneath the surface, with minimal thermal effects. Consequently, LSP has been shown to effectively inhibit fatigue cracking and SCC [42,43], as well as to improve resistance to uniform [36,44,45] and pitting corrosion [46,47] in various materials. Despite these advantages, the mechanisms by which LSP influences the passive film characteristics of AM Ti–6Al–4V alloys remain poorly understood.

Because the corrosion resistance of Ti alloys is primarily controlled by the stability and electrochemical properties of the passive oxide film, understanding the passivation behavior of Ti–6Al–4V alloys is essential for clarifying the corrosion mechanisms of AM materials. The passivation behavior of Ti–6Al–4V alloys is governed by the properties of the TiO_2_-based passive film, which behaves as an n-type semiconductor in which oxygen vacancies act as electron donors [48,49]. Mott–Schottky studies on SM Ti–6Al–4V have reported donor densities on the order of 10^20^ m^−3^, varying with film formation potential [50]. In AM Ti–6Al–4V alloys, however, the dominance of metastable α′ martensite and reduced β-phase content result in a thinner and less stable passive film, associated with higher passive current densities and greater susceptibility to film dissolution [51,52,53,54]. These findings underscore that passive film breakdown—serving as the precursor to both pitting corrosion and SCC—is critically sensitive to the AM-induced microstructure, highlighting the importance of systematically investigating passivation behavior in AM Ti–6Al–4V alloys.

The corrosion resistance of passive alloys is influenced not only by macroscopic defects such as pores and voids but also by the intrinsic stability of the passive film. According to the point defect model [55], the stability and localized failure of passive films are determined by the formation, migration, and dissipation of point defects within the film. Because point defect characteristics govern the semiconducting behavior of passive films, Mott–Schottky analysis has been widely employed to evaluate the electrochemical stability of passive alloys, particularly in SM materials [56,57].

Although recent years have witnessed increasing research on the corrosion behavior of AM alloys, studies on the point defect characteristics of AM passive films remain limited. Existing work has primarily focused on stainless steel, Ti–6Al–4V, and Ni-based alloys [58,59,60], with findings suggesting that AM alloys exhibit a higher density of point defects than their SM counterparts. This increase in defect concentration has been linked to higher passive current density and greater susceptibility to pitting corrosion [58,59]. Despite these insights, there exists an urgent need to comprehensively examine point defects in AM Ti–6Al–4V passive films to elucidate the impact of AM processing on the corrosion mechanisms of passive metals and alloys [60].

However, directly examining the effects of point defects in AM passive films remains challenging, as traditional bulk polarization techniques provide limited insight into the localized evolution of point defects in microstructurally complex alloys. Recently, advanced electrochemical techniques, such as dynamic electrochemical impedance spectroscopy, the scanning vibrating electrode technique, and scanning electrochemical microscopy, have been applied to investigate localized electrochemical activity and passive film heterogeneity at the microscale [61,62]. While these advanced techniques provide valuable insight into the nanoscale distribution of defects, they do not directly quantify the overall defect concentration or charge transport mechanisms within passive films. Therefore, complementary electrochemical approaches, particularly Mott–Schottky analysis, remain essential for evaluating the semiconductor properties of passive films and for understanding their passivation behavior.

Considering these aspects, this study clarifies the passivation behavior of AM Ti–6Al–4V alloys from a physicochemical perspective by linking the point defect characteristics of passive films with microstructural and electrochemical responses. To this end, the point defect properties of passive films formed on AM alloys were systematically investigated and directly compared with those of conventionally wrought (SM) alloys. In addition, the effects of LSP on the microstructure and electrochemical behavior of both alloys were examined. This approach provides new insight into the role of AM-induced microstructures in governing passivity and defect chemistry in Ti–6Al–4V alloys.

## 2. Experimental Procedures

### 2.1. Sample Preparation

SM Ti–6Al–4V alloy sheets and AM Ti–6Al–4V bars were used as the experimental specimens. The SM material was a commercially available hot-rolled Ti–6Al–4V plate with a nominal Ti–6Al–4V composition (Al ≈ 6 wt.%, V ≈ 4 wt.%). The AM Ti–6Al–4V alloy was fabricated via direct energy deposition (DED) (InssTek, Inc., Daejeon, Republic of Korea). The chemical composition of the Ti–6Al–4V alloy used in the DED process was as follows: Ti bal., C 0.01%, O 0.13%, N 0.02%, H 0.002%, Fe 0.2%, Al 6.62%, and V 4%, with each other element < 0.1%. The total content of other elements was below 0.4%. The DED process was performed using a beam size of 1200 μm and laser power of 500–812 W. The powder feeding rate was maintained at 3 g·min^−1^, and the carrier gas flow rate (Ar) was set as 3.4 L·min^−1^. The tool path followed a zigzag pattern. The specimens were cut into pieces sized 50 mm × 35 mm × 2 mm. The AM test pieces were extracted along the planes perpendicular to the build direction (BD specimens) and transverse to the build plane (TD specimen).

### 2.2. LSP Sample Preparation

LSP samples were prepared using laser pulses generated from the first stage of the power amplifier of a Bivoj Laser, a Yb:YAG laser system operating at a wavelength of 1030 nm and repetition rate of 10 Hz [63]. The pulses had a duration of 10 ns with a rectangular temporal profile. Prior to the treatment, the samples were covered with a 100-μm-thick black vinyl tape to protect the surface against thermal effects. Laminar water flow (approximately 1 mm) was applied to confine the plasma to the sample. Laser pulses with an energy of 2 J were focused onto a 2 mm laser spot, resulting in a specific energy density (fluence) of 63.7 J·cm^−2^ and power density of 5 GW·cm^−2^. To prevent the tape from tearing, the pulses were delivered in a layer consisting of four sequences. Each sequence adopted a laser pattern with 0% overlap between individual laser spots, and successive sequences were shifted by 1 mm in the *X*, *Y*, and *XY* directions on the exposed surface to achieve a 50% laser spot overlap within the treated layer.

### 2.3. Microstructure Analysis and Hardness Measurement

To examine the alloy microstructure, specimens were ground using SiC papers up to 4000 grit and finished with a 1 μm alumina paste. The prepared specimens were observed using optical microscopy (OM, AMIO, ZEISS, Jena, Germany). For more detailed microstructural characterization, scanning electron microscopy (SEM, SU-8600, Hitachi, Tokyo, Japan) was conducted at an accelerating voltage of 15 kV to examine the microstructural changes induced by LSP in the SM and AM specimens. Before OM and SEM observation, the specimen surfaces were etched using Keller’s reagent for 30 s. Phase identification was performed using X-ray diffraction (XRD, Empyrean, Malvern Panalytical, Almelo, The Netherlands). Surface residual stresses in the LSP specimens were measured by XRD using the sin^2^ ψ method with a Rigaku AutoMATE II X-ray diffractometer (Tokyo, Japan) and Cr Kα radiation (*λ* = 2.2897 Å). These measurements were performed in two perpendicular directions relative to the laser peening direction.

The surface hardness was measured using a Vickers hardness tester (HMV-G, Shimadzu Corporation, Kyoto, Japan) with a load of 2 kgf applied for 10 s at five points on each sample surface. Surface roughness measurements (SJ-210, Mitutoyo, Kawasaki, Japan) were performed using a contact-type roughness meter along five randomly selected lines (length of 6 mm) on the surface.

### 2.4. Electrochemical Tests

Potentiodynamic polarization, potentiostatic polarization, and Mott–Schottky tests were performed sequentially using a potentiostat (VMP-3, Bio-Logic, Seyssinet-Pariset, France). Prior to electrochemical testing, the working electrodes were prepared as follows: Unpeened specimens were ground using SiC paper up to 2000 grit, rinsed with distilled water and ethanol, and dried in air. LSP specimens were used as is, without polishing, to preserve the shock-induced surface morphology. All specimens were mounted in epoxy resin, leaving an exposed surface area of 0.5 cm^2^. All current densities were normalized to this geometric surface area without surface roughness correction. A high-density carbon rod and saturated calomel electrode (SCE) were used as the counter and reference electrodes, respectively. The electrolyte was a pH 2 buffer solution (18 ± 2 °C) that was degassed with ultrapure nitrogen gas for 1 h. This solution was prepared by mixing 975 mL of solution 1 (0.2 M H_3_BO_3_ + 0.05 M H_8_C_6_O_7_·H_2_O) with 25 mL of solution 2 (0.1 M NaPO_4_·12H_2_O). This acidic condition was chosen because it promotes partial dissolution of the passive film on Ti, enabling more sensitive evaluation of LSP-induced changes in passivation behavior.

After stabilization at the open circuit potential for 1800 s, potentiodynamic polarization was performed from −1.0 *V*_SCE_ to 1.3 *V*_SCE_ at a scan rate of 1 mV·s^−1^. Upon reaching 1.3 *V*_SCE_, potentiodynamic polarization was performed at this potential for 24 h to allow the passivation film to stabilize. Mott–Schottky analysis was then performed using staircase potentio-electrochemical impedance spectroscopy. The potential was decreased from 1.3 *V*_SCE_ to −1.0 *V*_SCE_ at a rate of approximately 0.1 V·s^−1^. At each potential, the system was stabilized for 5 s before measurement. An AC amplitude of 10 mV and a frequency of 1 kHz were applied during the measurements.

## 3. Results and Discussion

### 3.1. Microstructure, Hardness, and Residual Stress Characterization

**Figure 1:**
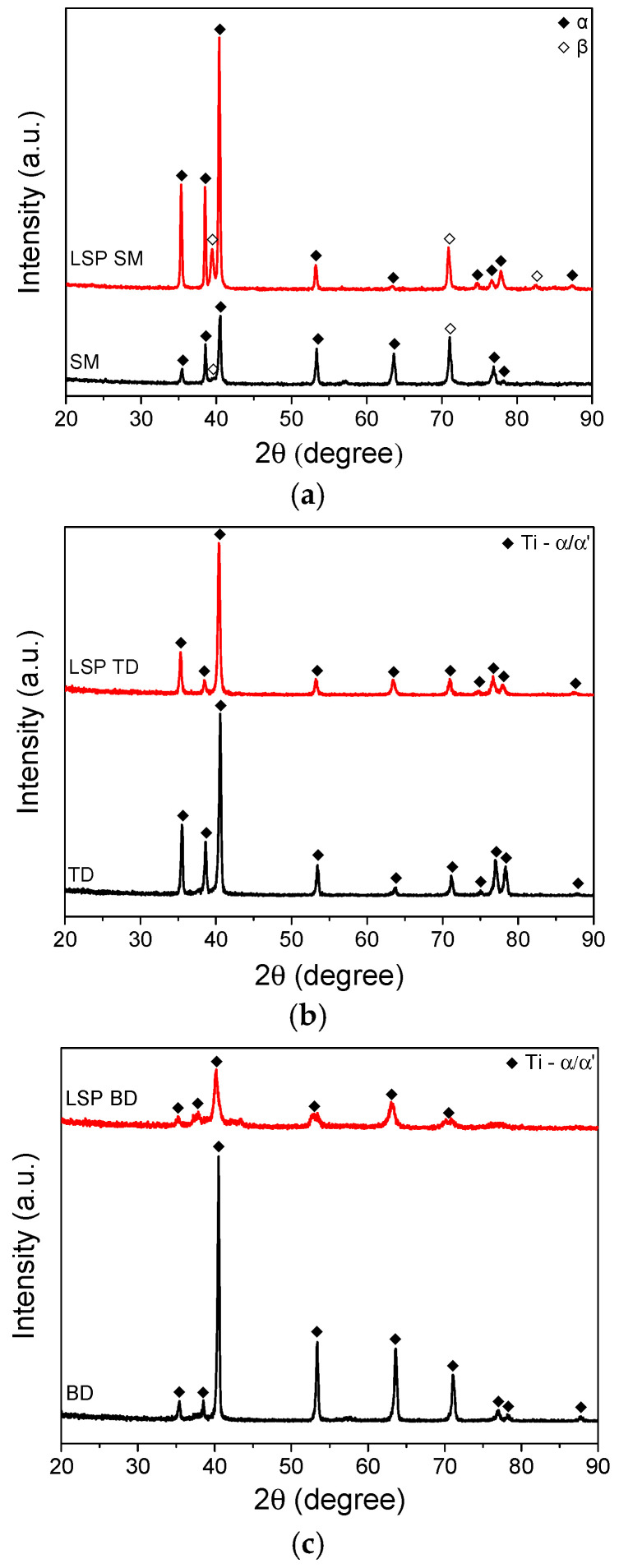
XRD patterns of (**a**) SM, (**b**) TD, and (**c**) BD specimens before and after LSP.

Figure 1 shows the XRD patterns obtained from the specimen surface before and after LSP treatment. For the SM specimen (Figure 1a), diffraction peaks corresponding to the α and β phases were observed. After LSP, the intensity of the β (110) peak at 39.5° increased, and a β (220) peak appeared at 82.6°. The increase in β phase intensity may be associated with stress–induced α→β phase transformation, in which the accumulated compressive stress is partially relaxed through the formation of the β phase. The β phase has more active slip systems than the α phase, enabling more effective accommodation of the LSP-induced deformation [64]. For the AM specimens (Figure 1b,c), only α and martensitic α′ phase peaks were identified both before and after LSP.

**Figure 2:**
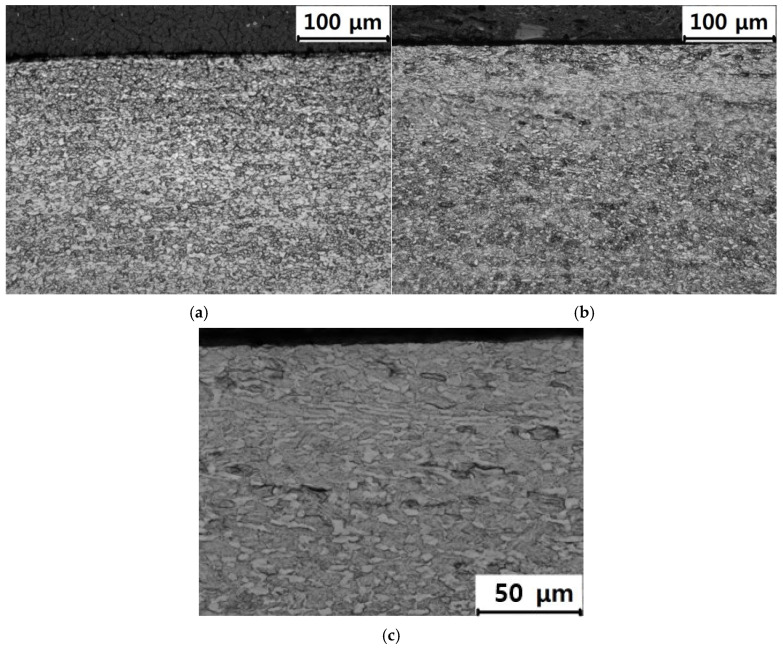
Cross-sectional microstructures of (**a**) unpeened SM Ti–6Al–4V (×200), (**b**) SM Ti–6Al–4V with LSP (×200), and (**c**) SM Ti–6Al–4V with LSP (×500).

**Figure 3:**
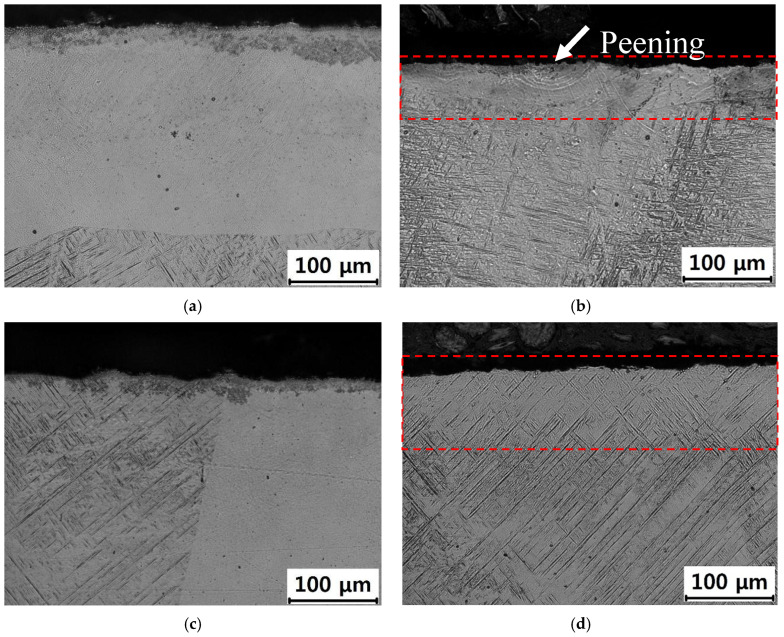
Cross-sectional microstructures of (**a**) Ti–6Al–4V TD, (**b**) LSP Ti–6Al–4V TD, (**c**) Ti–6Al–4V BD, and (**d**) LSP Ti–6Al–4V BD specimens.

**Figure 4:**
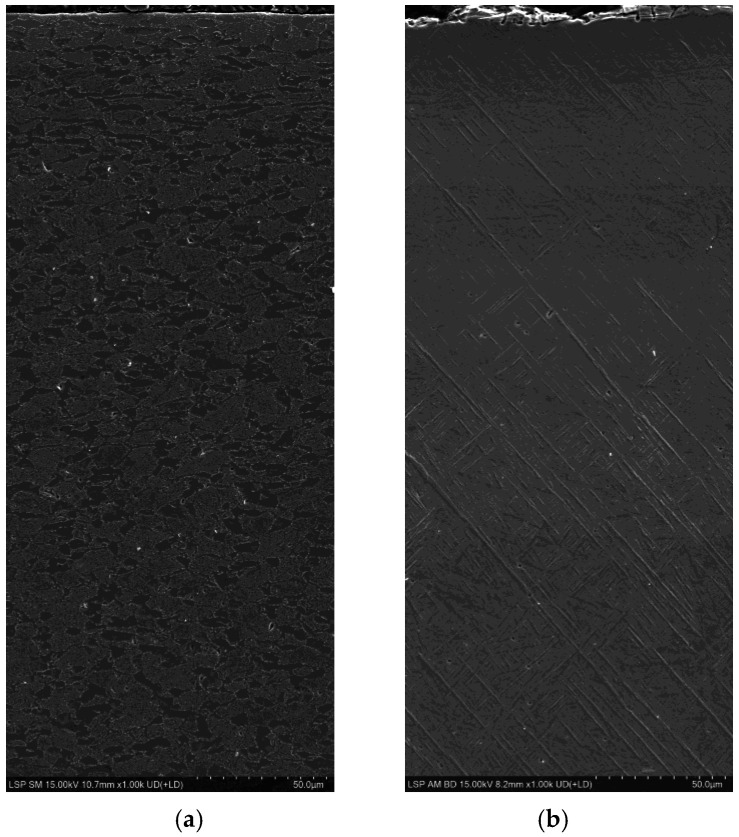
SEM microstructures showing the microstructural changes induced by LSP in (**a**) SM and (**b**) AM TD specimens.

The cross-sectional microstructures observed in the SM and AM alloys using OM are presented in Figure 2 and Figure 3, respectively. The SM alloys (Figure 2a,b) consisted of α + β phases, consistent with the XRD patterns (Figure 1a). After LSP (Figure 2b), the grains near the surface appeared to be deformed and refined compared with those in the unpeened sample (Figure 2a). Grains in the region extending from the surface to 40 μm were finer than those in deeper regions. In the region between 30 μm and 50 μm, the grains appeared severely deformed, whereas below 60 μm, the microstructure was similar to that of the unpeened specimen. Grain refinement has been commonly observed in shot peening [65,66,67] and LSP processes [36,68,69]. A previous study [70] reported that the average size of the α phase decreased from approximately 10 μm to 0.5 μm after LSP. In the present study, the grain size of the unpeened Ti–6Al–4V alloy was 5–8 μm, which, after LSP, decreased to 3–4 μm in the region extending from the surface to 40 μm. At a depth of 40–60 μm, the deformed grains exhibited horizontal and vertical dimensions of 15–25 μm and 2–3 μm, respectively. To further investigate the LSP-induced microstructural changes observed by OM, SEM observations were conducted for the LSP-treated SM and AM TD specimens. The corresponding images are presented in Figure 4. As shown in Figure 4a, the LSP-treated SM specimen exhibited a more refined and compressed microstructure near the surface compared with that in deeper regions, consistent with the grain refinement observed by OM.

In the case of the AM Ti–6Al–4V alloy, both the BD and TD specimens showed alternating light and dark regions containing the acicular martensite α’ phase (Figure 3). Such light and dark areas are commonly observed in AM Ti alloys, attributable to the rapid cooling and heating cycles involved in the DED process [71]. Regions that experience different cooling rates develop distinct microstructures, resulting in noticeable differences in appearance. Additionally, process parameters such as laser power, scanning speed, and layer thickness influence the thermal history of the material, thereby affecting the microstructural evolution and contributing to the formation of light and dark regions. In this study, LSP treatment resulted in shockwave marks sized several tens of micrometers (Figure 3b) with a spacing of 1 mm. The acicular α’ phase was partially fragmented and eliminated by LSP, with this effect being more pronounced near the shockwave marks (Figure 3b) and in the shallow subsurface region (Figure 3d). These changes could not be detected by XRD (Figure 1b,c) as this technique cannot distinguish between the α and α’ phases owing to their identical hexagonal close-packed (HCP) crystal structure. The strong shockwaves of LSP are thus thought to fragment the brittle α’ phase or cause stress-indued phase transformation. For the AM TD specimen, SEM observation of the LSP sample (Figure 4b) confirmed the partial disappearance and fragmentation of the acicular martensitic α′ phase from the surface to a depth of approximately 100 μm, consistent with the OM results.

**Figure 5:**
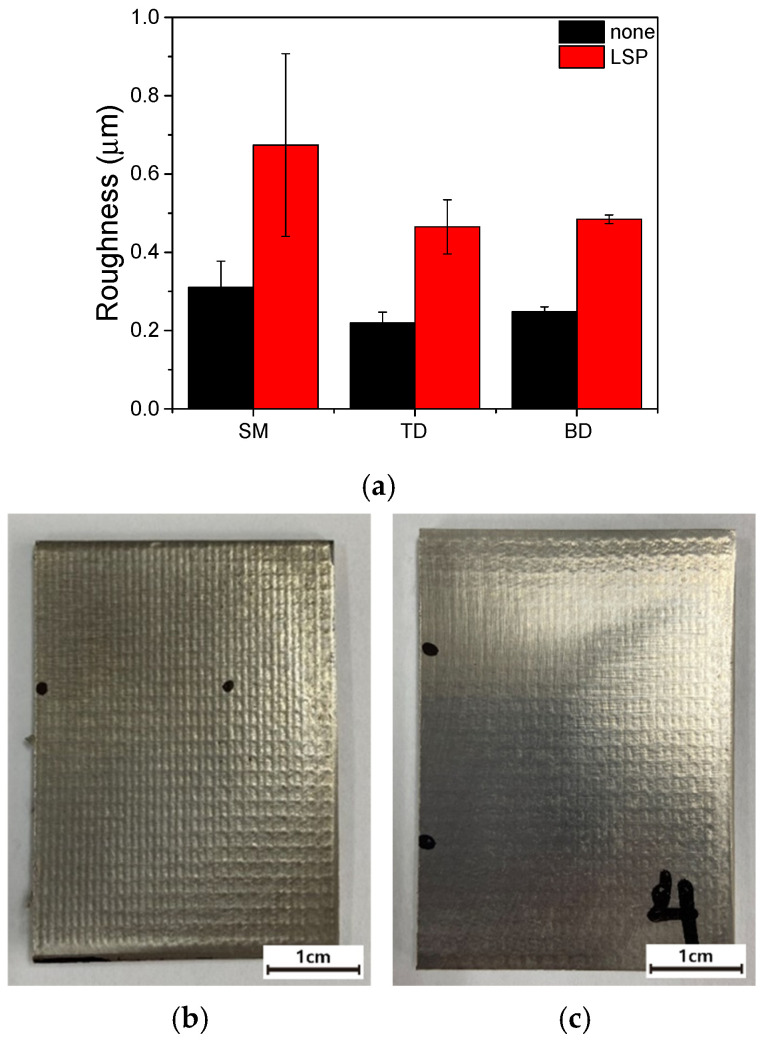
(**a**) Surface roughness (*Ra*) of the LSP and unpeened samples. (**b**) Macro-image of the LSP SM sample. (**c**) Macro-image of the LSP AM sample.

LSP resulted in an increase in surface roughness (*Ra*), as shown in Figure 5. Specifically, the *Ra* of the LSP-treated samples was 0.5–0.7 µm, more than twice those of the unpeened specimens (0.2–0.3 µm). This increase in roughness at the macroscale is attributable to the periodic indentations produced at the shock impact points, as shown in Figure 5b. Nevertheless, no significant changes in the surface profiles were observed at the microscopic scale (Figure 2 and Figure 3).

**Figure 6:**
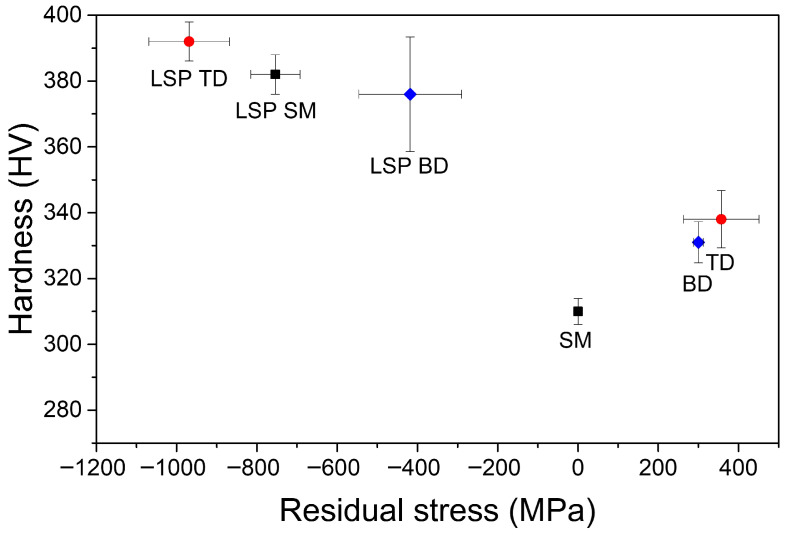
Effects of LSP on surface hardness and surface residual stress in SM and AM Ti–6Al–4V alloys.

As expected, LSP treatment resulted in an increase in surface hardness (Figure 6, *y*-axis). The hardness of the unpeened specimens was 310–340 HV, whereas that of the LSP specimens was 370–390 HV. In general, LSP results in a high density of dislocations near the surface, leading to microstructural refinement and increased hardness [39,72,73,74]. The hardness increment observed in the present study was 12–16%, consistent with the work of Nalla et al. [75], who reported a hardness increase exceeding 10% following LSP.

This increase in hardness is associated with the effects of the residual stress [76], grain size, and dislocation concentration. Figure 6 shows that the hardness increased with increasing residual stress, regardless of whether the residual stress was tensile or compressive. All LSP specimens showed a high compressive residual stress of 400–950 MPa. In contrast, the AM specimens initially exhibited tensile residual stress of approximately 300 MPa before the LSP treatment (Figure 6, *x*-axis). The tensile residual stress in AM samples is mainly ascribed to the shrinkage of the material as the melt pool solidifies [77,78]. These results demonstrate that LSP is highly effective in inducing compressive stress in materials, consistent with numerous reports that have highlighted improvements in cracking and fatigue resistance following LSP [2,36,40,42,46,72,73,74,79].

The compressive residual stress in the SM specimen after LSP was not greater than that in the AM specimens. Notably, the SM alloy did not exhibit tensile residual stress before LSP. Additionally, the compressive residual stress in the TD specimen after LSP was considerably higher than that of the BD specimen, even though both specimens exhibited similar tensile stress values before LSP. These results indicate that LSP treatment is highly effective in alleviating tensile stress. However, the magnitude of the compressive stress retained after LSP does not depend solely on the initial stress state. Instead, the surface phases (light and dark regions in Figure 3) and plane direction relative to the build direction influence the effectiveness of the LSP in inducing compressive stress.

### 3.2. Electrochemical Characterization

**Figure 7:**
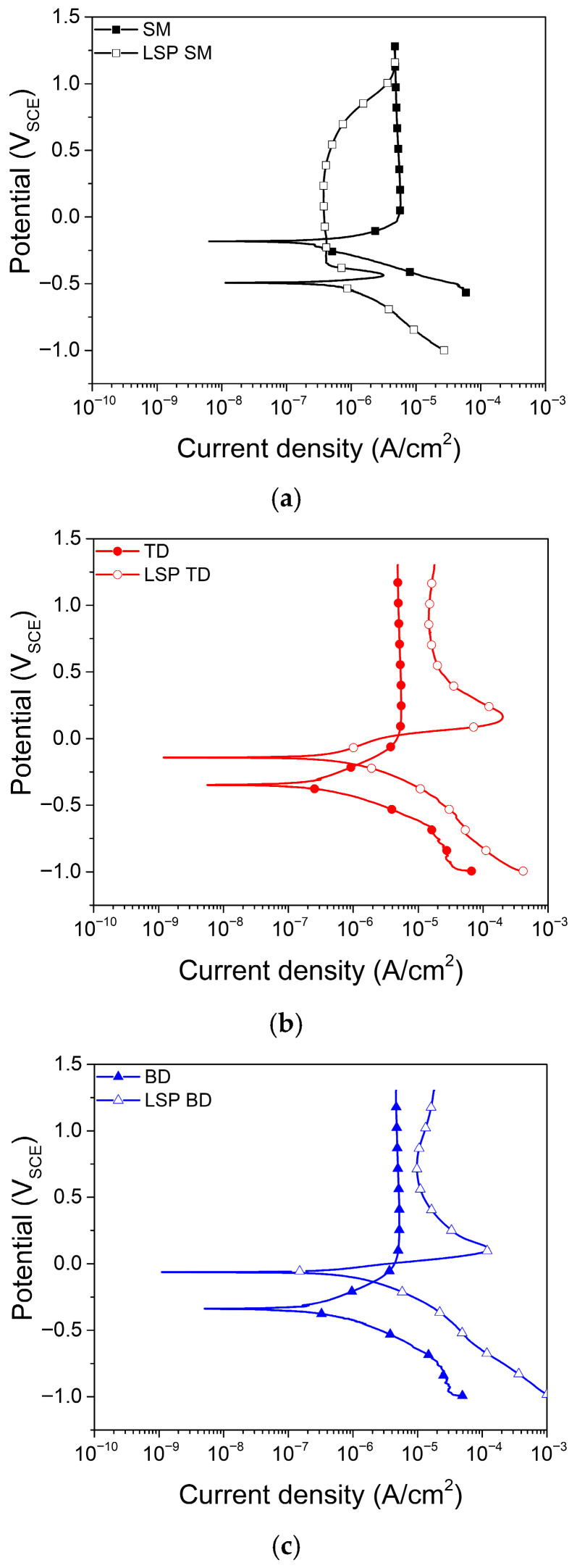
Potentiodynamic polarization curves for (**a**) SM Ti–6Al–4V, (**b**) AM Ti–6Al–4V (TD), and (**c**) AM Ti–6Al–4V (BD) alloys before and after LSP, measured in a pH 2 buffer solution.

Figure 7 presents the electrochemical polarization curves of the SM and AM Ti–6Al–4V alloys measured in a pH 2 buffer solution before and after LSP. The unpeened SM alloy showed highly stable passive behavior, with a passive current density below 10^−5^ A·cm^−2^ and no anodic current peaks. The TD and BD AM alloys also showed passive behavior similar to that of the SM alloy, except for the value of the corrosion potential.

All LSP-treated specimens (SM, TD, and BD) exhibited an active–passive transition, whereas the unpeened specimens remained in the passive region. The critical anodic current density of the LSP SM specimen was approximately 3 × 10^−6^ A·cm^−2^, and its passive current density was lower than 10^−6^ A·cm^−2^ at potentials below 0.5 *V*_SCE_ (Figure 7a). According to the Pourbaix diagram [80], the anodic current peak at approximately −0.5 *V*_SCE_ is attributable to the oxidation of Ti^2+^ to Ti^3+^ which may become thermodynamically feasible under the influence of LSP-induced residual stress and lattice distortions. The anodic current peak at approximately 0–0.2 *V*_SCE_ observed in the AM alloys is attributed to the oxidation of V^3+^ to V^4+^ (VO^2+^), reflecting the dissolution of supersaturated V in the martensitic α’ phase [75]. This process is further promoted by LSP–induced residual stress and increased dislocation density, which act as diffusion pathways for metal-ion dissolution. At potentials above 0.5 *V*_SCE_, the passive current density increased in all LSP-treated specimens, corresponding to the oxidation of V^4+^ to V^5+^, as indicated by the Pourbaix diagram.

**Figure 8:**
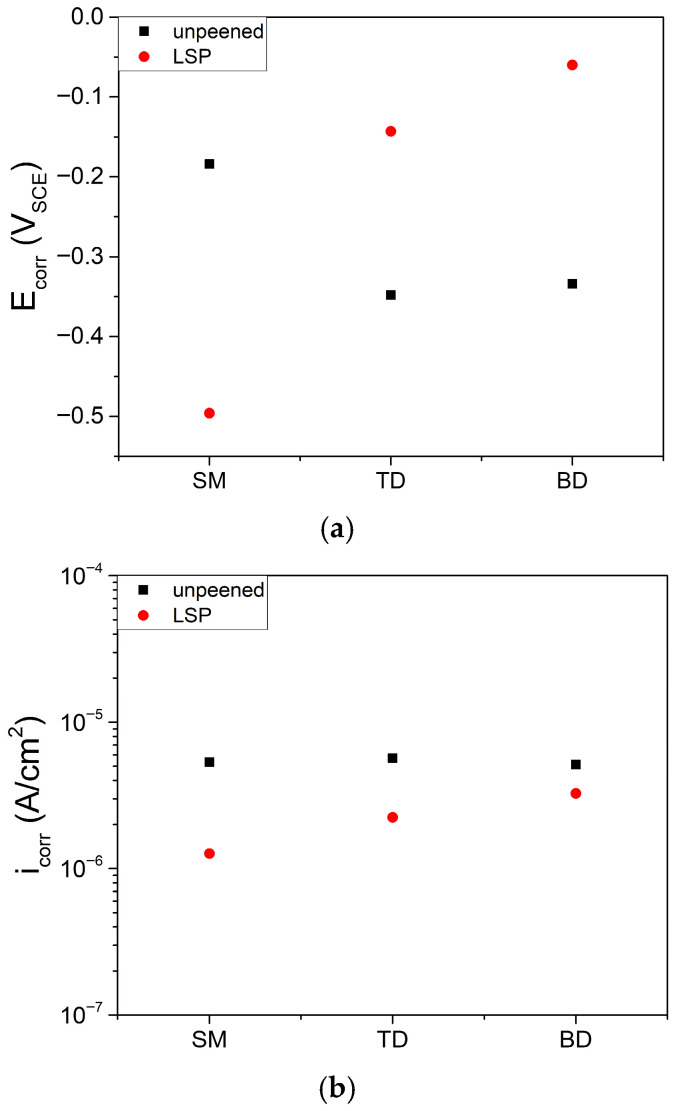
Corrosion (**a**) potential and (**b**) current density determined from potentiodynamic polarization curves. For LSP-treated specimens, *i*_corr_ was estimated by Tafel extrapolation. For unpeened specimens, the current density at 0.5 *V*_SCE_ was used as *i*_corr_.

In this study, *i*_corr_ was determined using different approaches depending on the electrochemical behavior of each specimen. For LSP-treated specimens, which exhibited a clear active–passive transition, *i*_corr_ was estimated by Tafel extrapolation. For unpeened specimens, which showed stable passive behavior without a distinct active region, the current density at 0.5 V_SCE_ was used as a measure of corrosion rate, as Tafel extrapolation was not applicable [81]. In the unpeened condition, the *i*_corr_ values of the AM specimens (TD and BD) were comparable to that of the unpeened SM specimen, all being of the order of 5 × 10^−6^ A·cm^−2^. After LSP treatment, *i*_corr_ decreased for all specimens and remained of the order of ~10^−6^ A·cm^−2^ (Figure 8b). This reduction in *i*_corr_ is closely associated with the introduction of compressive residual stress by LSP process, which has been widely reported to suppress anodic dissolution and localized corrosion processes by reducing the effective driving force for corrosion reactions [82].

In the case of the AM alloy, although the LSP-treated specimens exhibited slightly higher current densities than the unpeened specimens in the passive region above the corrosion potential (E_corr_), this behavior did not considerably influence the initial *i*_corr_ value. This observation suggests that the inhibitory effect of compressive residual stress on early-stage anodic dissolution outweighs potential adverse contributions from localized V dissolution.

Previous studies have reported that increasing the β-phase fraction enhances the corrosion resistance of Ti–6Al–4V alloys, whereas the martensitic α′ phase is generally more susceptible to corrosion [51,52,53,83]. In the present study, despite partial decomposition of the martensitic α′ phase induced by LSP (Figure 3 and Figure 4b, and Section 3.1), the *i*_corr_ value was observed to decrease.

The surface roughness after LSP increased by approximately twofold, as shown in Figure 5a and discussed in Section 3.1. Although increased surface roughness is generally associated with accelerated corrosion [84,85,86,87], the *i*_corr_ in the present study either decreased or remained nearly unchanged after LSP. While direct quantitative comparison with results obtained in different environments is not feasible, these findings highlight that the detrimental effect associated with increased surface roughness could be offset by LSP-induced factors such as compressive residual stress and limited β-phase formation.

**Figure 9:**
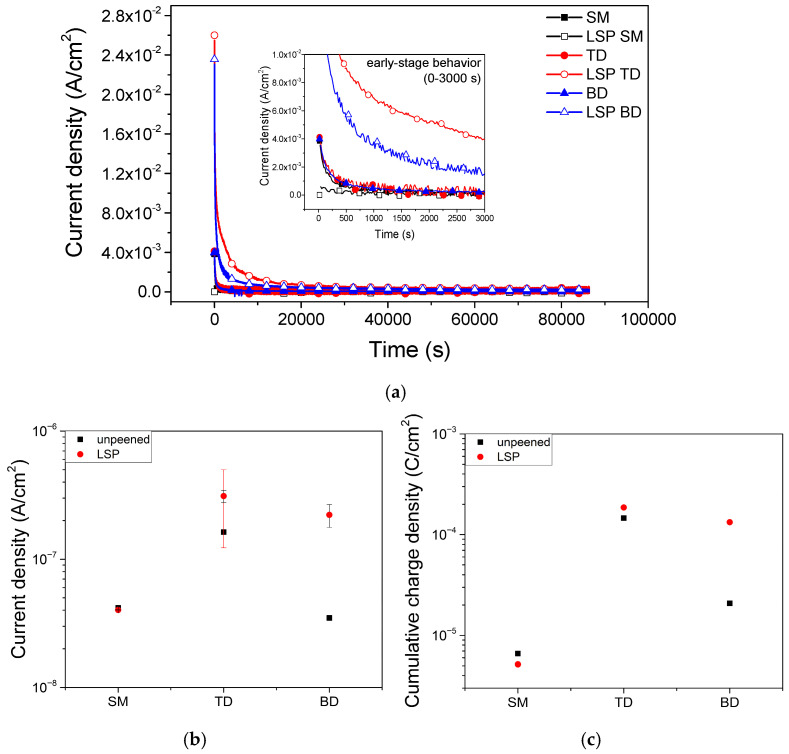
(**a**) Passive current density transient at 1.3 *V*_SCE_ for 24 h, (**b**) average current density in the last 600 s, and (**c**) cumulative charge density integrated over the last 600 s in a pH 2 buffer solution.

At 1.3 *V*_SCE_, 24 h potentiostatic polarization (Figure 9a) showed that the current density decreased over time and approached a steady-state value. The LSP AM and LSP SM specimens displayed the highest and lowest initial current densities, respectively. This trend is consistent with the final passive current density (*i*_pass_) obtained from the potentiodynamic polarization curves (Figure 7). After 24 h, the unpeened SM, LSP SM, and unpeened AM samples reached steady-state passive current densities (*i*_ss_) within the 10^−8^–10^−7^ A·cm^−2^ range. The LSP AM specimen retained a higher *i*_ss_ than its unpeened counterpart.

This electrochemical behavior correlates well with the microstructures. In the SM alloy, LSP led to grain refinement and a slight increase in the β phase (Figure 1a and Figure 2b). This refined microstructure accelerated the initial formation of the passive film, as reflected by the decreased *i*_ss_ at early stages (Figure 9a). However, *i*_ss_ changed little after 24 h (Figure 9b), indicating that although microstructural refinement accelerates early passivation, it exerts a limited impact on the long-term stability of the passive film.

In contrast, the AM alloy was dominated by a martensitic α′ phase with notable build-layer boundaries and pores (Figure 1b,c, Figure 3 and Figure 4b). Zhan et al. [54] reported that, owing to the presence of an α′ matrix, a minor β phase, and high porosity, laser powder bed fusion Ti–6Al–4V develops a thinner and less stable passive film than cast Ti–6Al–4V, resulting in a passivation current density approximately an order of magnitude higher. Consistent with these findings, the unpeened AM in the present study already exhibited higher *i*_ss_ than the SM specimens, and *i*_ss_ further increased after LSP. This behavior suggests that α′-phase-dominated microstructures promote the formation of defect-rich passive films with reduced long-term stability.

The cumulative charge density (*Q*_final_), shown in Figure 9c and calculated over the final 600 s of the 24 h potentiostatic polarization period, provides a quantitative measure of the electrochemical activity under near-steady-state conditions. Among the tested specimens, the LSP SM alloy exhibited a *Q*_final_ value of 5.17 × 10^−6^ C·cm^−2^, comparable to that of the unpeened SM (6.59 × 10^−6^ C·cm^−2^), indicating that LSP does not adversely affect the long-term passivation stability of the SM alloy and may slightly improve it. In contrast, the AM specimens showed substantially higher *Q*_final_ values, ranging from 2.08 × 10^−5^ C·cm^−2^ to 1.86 × 10^−4^ C·cm^−2^, reflecting increased charge consumption during the later stages of passivation. These results suggest that differences in cumulative charge behavior are associated with variations in microstructure and the resulting defect characteristics of the passive film.

**Figure 10:**
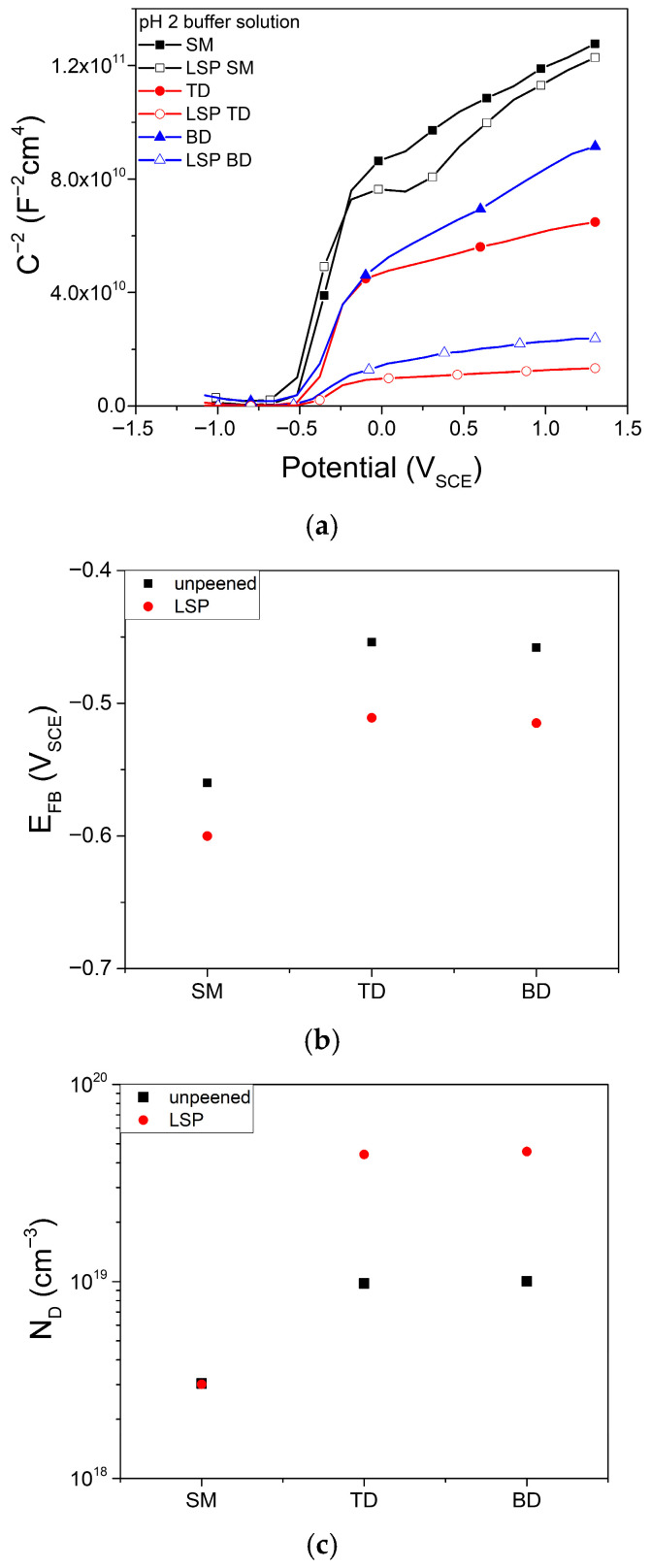
(**a**) Mott–Schottky plots, (**b**) donor concentration, and (**c**) flat-band potential of passive films on Ti-6A-4V alloys in a pH 2 buffer solution.

A Mott–Schottky analysis was performed to assess the semiconducting properties of the passive film. The Mott–Schottky plot of the film grown at 1.3 V for 24 h (Figure 10a) exhibits the characteristics of an n-type semiconductor, which is typical of passive films on Ti alloys [48,49]. For n-type semiconductors, the donor density (*N*_d_) and flat-band potential (*E*_FB_) can be obtained as

(1) $$\frac{1}{C^{2}}=\frac{2}{\varepsilon \varepsilon_{0}eN_{d}}(E-E_{FB}-\frac{kT}{e}),$$

where *C* is the capacitance of the space-charge layer; *ε* is the dielectric constant of the passive film, which is approximately 85 for a Ti alloy [50]; *ε*_0_ is the dielectric constant in vacuum (8.854 × 10^−14^ F·cm^−1^); *e* is the electric charge of an electron (1.602 × 1^−19^ C); *E* is the applied potential; *k* is the Boltzmann constant (1.38 × 10^−23^ J·K^−1^); and *T* is the temperature in K. It should be noted that the dielectric constant of Ti-based passive films can vary widely depending on film composition, structure and formation conditions [50], and therefore the calculated *N*_d_ values should be interpreted in a comparative sense rather than as absolute values.

*E*_FB_ decreased with LSP treatment (Figure 10b). In general, *E*_FB_ of TiO_2_ is influenced by the crystalline polymorph, crystalline orientation, and morphology, all of which can be influenced by the film synthesis or growth procedure [88]. According to the literature, *E*_FB_ of TiO_2_ in a pH 2 aqueous solution ranges from approximately −0.3 to 0 *V*_NHE_ and decreases with increasing anatase fraction. However, these data could not be directly compared with the present data owing to differences in the composition and surface conditions of the samples used in both studies.

*N*_d_, which represents the concentration of oxygen vacancies in n-type passive films, is shown in Figure 10c. The *N*_d_ values for the passive film on the SM alloys were lower than that for the AM alloys and were largely unaffected by LSP. In contrast, the *N*_d_ values of the AM alloys increased significantly after LSP treatment. The potentiodynamic polarization curves (Figure 7) imply that V dissolved in the form of VO^2+^ or VO_2_^+^, whereas Al did not undergo remarkable dissolution. This indicates that the passive film formed on the LSP-treated samples has fewer V^4+^ or V^5+^ ions compared with the film formed on the unpeened alloys. In general, ions with higher valence, such as V^4+^ and V^5+^, can reduce the concentration of oxygen vacancies (VO^2+^) in the passive film for charge balance if they substitute for Ti^4+^ in the TiO_2_ lattice. Therefore, the unpeened specimens, possibly with higher concentrations of V^4+^ and V^5+^ species, are likely to form less defective passive films.

The high concentration of point defects may contribute to the high passive current density observed for the LSP-treated alloys, as shown in Figure 9. However, *N*_d_ alone cannot explain the high current density. For example, although the unpeened BD alloy showed a current density comparable to that the SM alloys (Figure 9), its *N*_d_ was considerably larger, similar to that of the TD alloy. The passive current density is governed by the flux of point defects, which depends on both the concentration and diffusivity of these defects [89].

It is proposed that the increase in *N*_d_ after LSP in the AM alloys may be associated with the dissolution of V from the supersaturated α′ phase, as evidenced by the anodic peaks observed in the potentiodynamic polarization curves (Figure 7). The release of V species from the passive film may influence the defect structure of the film, potentially increasing the concentration of oxygen vacancies. According to the point defect model [55], an increase in oxygen vacancy concentration can enhance defect transport through the passive film, which may contribute to the reduced stability of the passive film. This interpretation is consistent with the higher iss and Q_final_ values observed in the LSP-treated AM specimens. However, it should be noted that this proposed mechanism is based on indirect electrochemical evidence, and direct verification of passive film composition, for example, by X-ray photoelectron spectroscopy, would be required to confirm this hypothesis.

Nevertheless, based on the general consistency in the trends of the passive current density in Figure 9 and *N*_d_ in Figure 10c, it can be inferred that a higher defect density leads to reduced protectiveness of the passive film. These results suggest that the diffusion coefficients of the vacancies in the passive films were not significantly changed by AM or LSP. Further work is required to elucidate the passivity of AM alloys under surface treatments, such as LSP, especially for BD samples that exhibit lower passive current densities than would be expected based on the *N*_d_ value.

## 4. Conclusions

The effects of LSP on the passivity of SM Ti–6Al–4V and AM Ti–6Al–4V alloys were investigated, and the following conclusions were derived.

1. LSP increased the surface roughness (*Ra* ≈ 0.25 to 0.6 μm), enhanced surface hardness by 12–16%, and introduced compressive residual stresses ranging from −450 to −950 MPa in both alloys. After LSP, the SM alloy exhibited β-phase enrichment and grain refinement, whereas the AM alloy showed a decrease in the α’ phase.
2. All specimens showed an active–passive transition after LSP, and the corrosion rate decreased from ~5 × 10^−6^ to ~1 × 10^−6^ A·cm^−2^. The introduction of compressive residual stress reduced the corrosion rate, despite the increased surface roughness.
3. During 24 h potentiostatic polarization at 1.3 *V*_SCE_, the current density of LSP AM specimens remained higher than that of their unpeened counterparts, whereas the LSP SM specimens showed current densities comparable to those of the other samples. These findings indicate that LSP effectively reduces the corrosion rate but may degrade the long-term passive film stability of AM Ti–6Al–4V alloys.
4. Potentiodynamic polarization results suggest that the behavior of SM and AM alloys is controlled by different anodic reactions. In the SM alloy, a rapid anodic process leads to a decrease in the corrosion potential, whereas in the AM alloy, a distinct anodic behavior delays passivation and increases the corrosion potential.
5. According to the Mott–Schottky analysis, the donor density of the passive films on SM specimens was lower than that of the AM specimens and changed marginally after LSP. In contrast, the donor density of the AM specimens was significantly increased by LSP, which increased the number of oxygen vacancies and decreased the stability of the passive films.

## Data Availability

The original contributions presented in this study are included in the article. Further inquiries can be directed to the corresponding author.
